# Choroidal Structure after Half-Dose Photodynamic Therapy in Chronic Central Serous Chorioretinopathy

**DOI:** 10.3390/jcm9092734

**Published:** 2020-08-24

**Authors:** Camilla Alovisi, Felice Cardillo Piccolino, Marco Nassisi, Chiara M. Eandi

**Affiliations:** 1 Department of Surgical Science, University of Torino, 10126 Torino, Italy; camillaalovisi@gmail.com; 2 Fondazione per la Macula Onlus, 16121 Genova, Italy; felice.cardillopiccolino@gmail.com; 3 Department of Clinical Sciences and Community Health, University of Milan, 20122 Milan, Italy; m.nassisi@gmail.com; 4 Ophthalmological Unit, Fondazione IRCCS Ca’ Granda, Ospedale Maggiore Policlinico, 20122 Milan, Italy; 5 Department of Ophthalmology, University of Lausanne, Jules-Gonin Eye Hospital, Fondation Asile des Aveugles, 1004 Lausanne, Switzerland

**Keywords:** choroid, choriocapillary, central serous chorioretinopathy, optical coherence tomography, OCT angiography, photodynamic therapy

## Abstract

The study aims to analyze the changes produced by half-dose photodynamic therapy (HD-PDT) in the choroid of eyes with chronic central serous chorioretinopathy (CSC) applying the binarization method to spectral domain optical coherence tomography (SDOCT) and OCT Angiography (OCTA) images. SDOCT and OCTA were performed before, one hour, one week, and one month after HD-PDT. Binarization with a modified Niblack method and analysis by ImageJ were applied. An average ratio between luminal part and total structure was calculated. Twenty-two eyes of 21 patients (20 male and 1 female; mean age 54.8 years) were enrolled. A statistically significant reduction of the central choroidal thickness was observed one week (from 407 µm to 362 µm, *p* = 0.034) and one month (from 407 µm to 341.5 µm, *p* = 0.0004) after HD-PDT. The baseline average ratio between luminal part and total structure was 33.4% in SDOCT, and 61.1% in OCTA. These values were 35.3% and 61% one hour, 33.9% and 60.4% one week, and 34.5% and 60.6% one month after HD-PDT, respectively. Overall, PDT seems to produce short-term changes on the luminal component of both choriocapillaris and choroid, which return to baseline status after one month from treatment. However, choroid stays significantly thinner after one month, with both luminal and interstitial components significantly reduced.

## 1. Introduction

Central serous chorioretinopathy (CSC) is a chorioretinal disease characterized by the accumulation of fluid under the neurosensory retina through a defect of the retinal pigment epithelium (RPE) that often resolves spontaneously. However, recurrences and chronic forms develop in nearly 50% of cases, leading to a progressive central vision impairment, caused by persistent subretinal fluid and extensive RPE and outer retinal dystrophic/atrophic changes [1,2]. Although the clinical findings are well known, the underlying pathogenetic mechanism is still unclear. A large number of studies suggest that in patients with CSC the choroid is primarily affected, and that the RPE is only secondarily involved [3].

The choroid, a thin-pigmented membranous layer lying underneath the Bruch’s membrane, is a highly vascular tissue that supplies oxygen and nutrients to the outer retina and is often involved in the pathogenesis of several chorioretinal diseases, such as CSC [4]. The choroidal thickness varies in humans from 100 µm anteriorly to 220 µm posteriorly [5], and its vessels are branches and anastomosis deriving from the posterior ciliary arteries.

Vascular filling delay, vessel dilatation, multifocal areas of choroidal vascular hyperpermeability, and punctuated hyperfluorescent spots are clearly visible with indocyanine green angiography (ICGA) in CSC [3,6,7]. Moreover, enhanced depth imaging (EDI) spectral domain optical coherence tomography (SDOCT) reveals increased choroidal thickness and dilated deep choroidal vessels (termed pachychoroid) [8], while swept-source OCT evidences a choriocapillary thinning [9].

Photodynamic therapy (PDT) represents a widely accepted treatment for chronic CSC as its efficacy—particularly with the half-dose and the half-fluence settings—has been proved by numerous reports [10,11,12]. However, its mechanism of action remains unclear, the main hypothesis is that PDT exerts its effect on the choroid through short-term vascular occlusion at the level of the choriocapillary (CC) and long-term remodeling of the congested choroidal vessels [13]. Direct consequence of this would be the normalization of the vascular caliber, with the reduction of the vascular hyperpermeability and leakage. Imaging techniques, such as ICGA and histological findings on human eyes with neovascular age-related macular degeneration, have demonstrated vascular occlusion affecting the CC few days after PDT [14,15,16].

Recently, SDOCT and OCT Angiography (OCTA) are allowing for new insights in the investigation of vascular and circulatory conditions in different chorioretinal disorders, including CSC [9,17,18,19]. In particular, OCTA is a dye-free technique that is able to analyze separately the different retinal vascular layers, the CC, and possible associated neovascular lesions [20]. OCTA findings in chronic CSC include increased blood flow, as well as flow voids in the CC, dilated choroidal vessels, and a type 1 choroidal neovascularization (CNV), that is not detectable with other imaging techniques [21,22]. Moreover, changes at the level of the CC have been reported after PDT in chronic CSC eyes [23].

In the present study, using a morphometric analysis of binarized structural OCT and OCTA images, we evaluated the choroidal vascular and stromal changes induced by half-dose photodynamic therapy (HD-PDT) giving further insights to its mechanisms of action in eyes with chronic CSC.

## 2. Methods

Imaging modalities and treatment are part of the normal clinical practice for CSC patients. Written informed consent was obtained from all the subjects prior to the examination and treatment. The data collection complies with Italian law. The Institutional Review Board approved the analysis of anonymized images from subjects with macular diseases in non-interventional studies (ASL TO2, n. 34/08/11). The study was conducted in accordance with the provisions stated in the Declaration of Helsinki (59th World Medical Association General Assembly; Seoul, Korea; October 2008).

### 2.1. Population

We prospectively included consecutive patients affected by chronic CSC lasting for more than three months referred to the University Eye Clinic in Torino from September 2016 to December 2017.

The exclusion criteria were eyes with other known ocular and retinal diseases, such as keratoconus, cataract, glaucoma, diabetic retinopathy, age-related macular degeneration, high myopia (more than −6.0 diopters), any other ocular diseases in which media opacities were present, and a presence of systemic disease like uncontrolled hypertension and diabetes. The presence of a pigment epithelium detachment was an exclusion criterion. Eyes with CSC treated with PDT or laser in the last six months or anti-VEGF therapies in the last three months were also excluded.

At baseline, all enrolled patients underwent the best correct visual acuity examination (BCVA) with ETDRS charts, anterior segment slit-lamp examination, and dilated fundus biomicroscopy. The intraocular pressure was measured in both eyes by applanation tonometry.

Angiography with fluorescein (FA) and indocyanine green (ICGA) (Heidelberg Spectralis, Heidelberg, Germany), SDOCT (Heidelberg Spectralis, Heidelberg, Germany) and OCTA using the split-spectrum amplitude-decorrelation angiography (SSADA) algorithm (RTVue XR Avanti with AngioVue; Optovue Inc., Fremont, CA, USA) were also performed by certified examiners after pupillary dilatation. ICGA guided HD-PDT with verteporfin was performed according to the procedure previously described [12]. SDOCT scans and OCTA images were obtained at baseline, one hour after the HD-PDT treatment, and also after one week and one month of follow-up.

### 2.2. Images Acquisition and Analysis

SDOCT and OCTA images were always taken from 9:00 AM to 11:00 AM in order to remove the confounding factor of the diurnal variations of the choroidal thickness.

SDOCT acquisition protocols included cross-section (single horizontal and vertical line) passing through the fovea, 30°, 768 A-scans, ART 100 frames, EDI mode, High Speed, and a macular volume scan EDI mode, 30° × 25°, 241 sections, 30 microns, ART 9 frames. The follow-up modality was active for the acquisition at the 1-hour, 1-week and 1-month visit. Central retinal thickness (CRT) was automatically measured in the central millimeter from the software of the machine (HEYEX, Heidelberg, Germany). Central choroidal thickness (CHT) was measured underneath the fovea from the outer surface of the hyperreflective RPE to the inner surface of the observed sclera. Two measurements were taken (on the central horizontal and vertical B-scans) and averaged. Two independent readers (CA and CME) measured the CHT in a masked fashion. Mean values were used for further analysis.

OCTA images were acquired using Optovue RTVue XR Avanti (Optovue Inc., Fremont, CA, USA) as previously reported [23]. At baseline, a 3 × 3 mm scan was centered on the area of leakage (as observed on the FA) or on the hyperpermeability area (as seen on ICGA) involving the fovea. During all post-treatment visits, the follow-up mode was used to ensure that all acquisitions were made in the exact same location. Only high-quality acquisitions (i.e., signal strength index >60 and no evident artifacts) were used for the study. The CC en-face angiograms were automatically generated by the instrument using a 30 µm-slab between 30 and 60 µm below the inner RPE reference. Two trained readers (CA and CME) independently checked the automated slab segmentation, performing a manual correction in case of inaccuracies. Discrepancies between readers were resolved by open adjudication. If no consensus was reached, a final decision was taken by a senior reader (FCP). All EDI-SDOCT and OCTA CC images were imported in ImageJ (version 1.49v; National Institutes of Health, Bethesda, Maryland, USA; [24]). Both images were locally thresholded using a modified Niblack method and analyzed as previously described [9,17]. In the EDI-SDOCT scans, a 1500 µm wide area under the fovea (750 µm nasal and 750 µm temporal to the center), extending vertically from the RPE to the chorioscleral border, was selected for the analysis. In the binarized EDI-SDOCT scans, the white zones corresponded to the interstitial spaces and the black zones to the lumen of vessels, while in OCTA images, it was the contrary. For each image, a trained ophthalmologist (CA) calculated the total area, the luminal area (LA) and the interstitial area (IA), then confirmed by a retinal specialist (CME). An average ratio between luminal part and total area in the CC (OCTA, equivalent to the vessel density in previous publications [23]) and in the whole choroid (SDOCT, equivalent to the choroidal vascularity index in previous publications [17,25]) was also calculated. Pre-treatment and post-treatment values were compared. Furthermore, for OCTA CC analysis, a comparison between the affected eye and the fellow healthy eye was performed.

### 2.3. Statistical Analyses

A commercial analytical package (Wizard version 1.9.15 for Mac) was used for the statistics. The Friedman test and the Wilcoxon signed-rank test were performed. Repeatability for CHT measurements was assessed using the intraclass correlation coefficient (ICC) with 95% confident interval (95% CI). Statistically significance was set at *p* value < 0.05.

## 3. Results

We analyzed 22 eyes of 21 patients (20 male; 1 female) affected by chronic CSC. Mean age was 54.8 years old (SD ± 9.2; range 51–59). Mean duration of symptoms was 21.5 months (SD ± 16.9, range 14–29). Five eyes had received a previous treatment at least six months prior to enrollment. In particular, two eyes underwent extra macular argon laser photocoagulation, and three eyes intravitreal injection of bevacizumab (Avastin, Genentech Inc, South San Francisco, CA). Mean BCVA was 0.35 ± 0.2 LogMAR (Snellen equivalent 20/40) at baseline, and it remained stable during follow-up.

Mean CRT was 324.5 ± 92.6 μm at baseline, 314.9 ± 69.3 μm at one hour, 275.4 ± 50.2 μm at one week, and 252.5 ± 50.1 μm at one month. CRT was significantly reduced at one week (Wilcoxon, *p* = 0.0002), and one month follow-up (Wilcoxon, *p* < 0.0001), but not at one hour (Wilcoxon, *p* = 0.28) when compared to baseline (Table 1).

Good agreement was observed for CHT measurements between the two operators (ICC: 0.966, 95% CI: 0.919–0.986). Mean CHT was 407.2 ± 97.2 μm at the baseline, 407.1 ± 103.6 μm at one hour, 362 ± 92 μm at one week, and 341.5 ± 98.2 μm at one month. Regarding CRT, subfoveal CHT showed a significant reduction from the baseline to one week (Wilcoxon, *p* = 0.034) and one month (Wilcoxon, *p* = 0.0004), but not one hour (Wilcoxon, *p* = 0.65) after the treatment (Table 1).

### 3.1. EDI-SDOCT Choroidal Area

The baseline mean choroidal area was 5996.811 ± 1631.5287 μm^2^, the LA was 2022.5354 ± 657.9681 μm^2^, and the IA was 3974.2755 ± 1007.2404 μm^2^.

One hour after HD-PDT the mean choroidal area was 5851.1718 ± 1625.2475 μm^2^, the LA was 2056.8678 ± 639.0139 μm^2^, and the IA was 3794.3039 ± 1025.0755 μm^2^.

One week after HD-PDT the mean choroidal area was 5902.6739 ± 1671.5518 μm^2^, with a LA and IA, respectively, of 2006.1363 ± 618.6754 μm^2^ and 3896.5376 ± 1079.9393 μm^2^.

**Figure 1:**
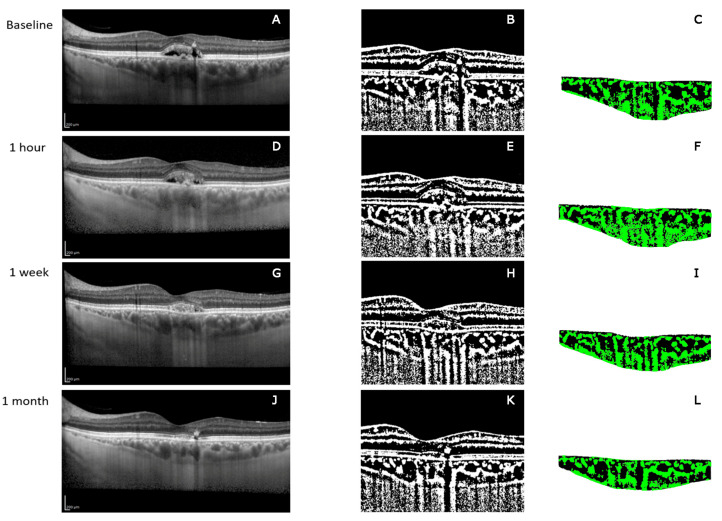
Enhanced depth imaging OCT (EDI-OCT) scans and converted binary images of a representative eye with chronic central serous chorioretinopathy (CSC) before and after half-dose photodynamic therapy (HD-PDT). EDI-OCT images (**A**,**D**,**G**,**J**) were converted to binary images (**B**,**E**,**H**,**K**) using the ImageJ software. In the selected 1500 µm wide area under the fovea (**C**,**F**,**I**,**L**), the luminal area (dark area) and the interstitial area (yellow area) can be seen and quantified. Binarization analyses show that the luminal area was reduced one week (**G**) and one month after HD-PDT (**J**). (**A**–**C**) Baseline; (**D**–**F**) one hour after HD-PDT; (**G**–**I**) one week after HD-PDT; (**J**–**L**) one month after HD-PDT.

At the one month visit after HD-PDT the mean choroidal area was 5736.5415 ± 1544.2734 μm^2^ with a LA of 1979.7304 ± 576.2866 μm^2^, and an IA of 3756.811 ± 1007.1412 μm^2^ (Figure 1).

Overall, the mean choroidal area was significantly different across the different follow-ups (Friedman test *p* = 0.006). A post-hoc analysis with Wilcoxon signed-rank test revealed that there was a significant difference between baseline and both one week and one month (*p* = 0.039 and *p* = 0.008 respectively) and between one week and one month (*p* = 0.013).

The ratio of the LA to the whole choroidal area was on average 33.4 ± 3%, 35.3 ± 3%, 33.9 ± 2.6%, and 34.5 ± 2.8% of the choroidal area at baseline, one hour, one week, and one month visit, respectively (Table 2). All the follow-up variations were statistically significant (Friedman test *p* = 0.0018). A post-hoc analysis with Wilcoxon signed-rank test revealed that there was a significant difference between the ratio of LA at baseline versus one hour (*p* = 0.013) and one hour versus one week (*p* = 0.20). However, there was not a statistically significant difference between the other follow-ups and baseline.

### 3.2. OCTA CC Area 

For the CC slab segmentation, there were no cases of disagreement between the readers.

At baseline, the mean LA was 5525.0509 ± 167.9515 μm^2^. One hour after HD-PDT the mean LA was 5521.4951 ± 159.6833 μm^2^, and reduced to 5460.1108 ± 120.9765 μm^2^ and 5480.0464 ± 133.3034 μm^2^ one week and one month after HD-PDT, respectively.

**Figure 2:**
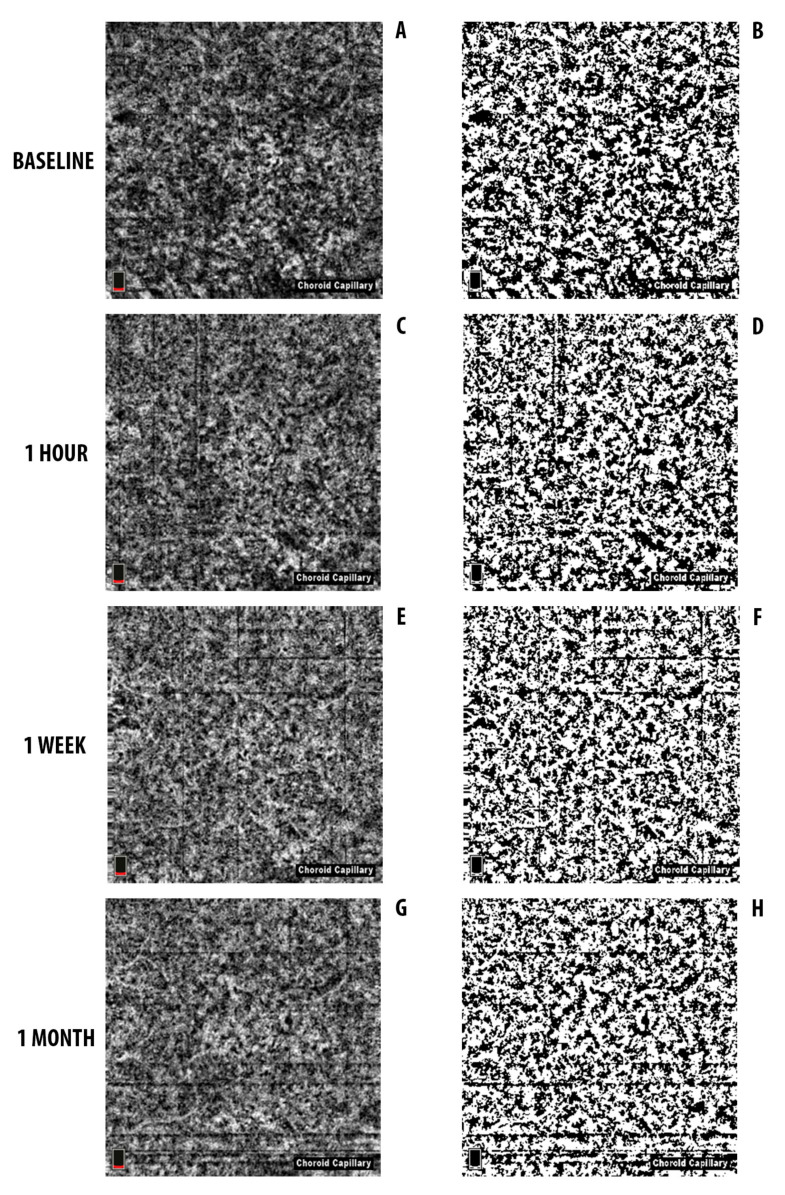
OCT angiography (OCTA) images and corresponding converted binary images of a representative eye with chronic central serous chorioretinopathy (CSC) before and after half-dose photodynamic therapy (HD-PDT) (same eye of Figure 1). OCTA images (**A**,**C**,**E**,**G**) were converted to binary images (**B**,**D**,**F**,**H**) using the ImageJ software. The luminal area (white area) and the interstitial area (dark area) are seen. Binarization analyses show that the ratio of luminal to the total choroidal area was reduced from 60.8% to 60.2% one week after HD-PDT (**E**,**F**), while returned to baseline value one month after HD-PDT (60.6%) (**G**,**H**). (**A**,**B**) Baseline; (**C**,**D**) one hour after HD-PDT; (**E**,**F**) one week after HD-PDT; (**G**,**H**) one month after HD-PDT.

The ratio of the LA to the total area of the CC was 61.1 ± 1.5%, 61 ± 1.8%, 60.4 ± 1.4%, and 60.6 ± 1.5% at baseline, one hour, one week, and one month visit, respectively (Table 3). All the follow-up variations were statistically significant (Friedman test *p* = 0.022) (Figure 2).

A post-hoc analysis revealed that there was a significant difference between one week and both baseline and one hour after treatment (*p* = 0.007 and *p* = 0.012 respectively).

The ratio of the LA to the total area of the CC was also calculated in the fellow healthy eye and was 60 ± 1%, 59.9 ± 0.8%, 60.1 ± 0.9%, 60 ± 1.2% at baseline, one hour, one week, and one month visit, respectively, and no statistically significant differences were found (Friedman test *p* = 0.174) (Table 3). In addition, the differences between the CSC eyes and the fellow eyes for the four visits were not statistically significant (Friedman test *p* = 0.32).

Finally, no statistically significant correlation at any time point was found in variations between CRT and LA and IA measured on both SDOCT and OCTA, while a statistically significant correlation was found between CHT and LA only at one month (coefficient Spearman *r* = 0.617, *p* = 0.002).

## 4. Discussion

In this paper, we report changes occurring in the choroid of eyes with chronic CSC following treatment with HD-PDT. With a binary image processing and quantitative analysis of SD-OCT B-scans and en-face OCT angiograms, we evaluated structural changes produced in the whole choroid and blood flow changes in the CC. We arbitrarily considered the blood flow signals of OCTA as an indicator of the vascular lumina of the CC. A similar morphometric analysis using the binarization method has been yet proposed to evaluate changes of the choroidal vascular structures following PDT in neovascular AMD [17].

Half-dose photodynamic therapy has previously proved to be effective in resolving leakage, and subsequently, subretinal fluid (SRF) in chronic CSC [12], although its mechanism of action has not been yet completely elucidated. It could involve damage of the CC leading to decrease of its hyperpermeability and resulting in cessation of leakage through the retinal pigment epithelium (RPE) [26,27,28].

In 1994 and 2002, Schmidt-Erfurth and Schlötzer-Schrehard described histopathological signs of choriocapillary damage and vascular remodeling one week after standard PDT in human eyes affected by age-related macular degeneration [16,17]. They found a complete obliteration of the choriocapillary vascular lumina by swollen endothelial cells, cell debris, fibrin and thrombocytes in the PDT-treated area. The overlying RPE and the photoreceptors showed no significant alterations. This choriocapillary shutdown corresponded to the hypoperfusion demonstrated by ICGA images acquired a few days after the treatment and reaching the maximal intensity after 3 to 7 days. The authors assumed that photoreceptors and RPE are able to tolerate a prolonged deprivation in oxygen supply, showing sometimes reduced function through a transient visual disturbance.

Recently the introduction of new non-invasive imaging techniques, such as EDI-SDOCT and OCTA, has improved the visualization of the chorioretinal vascular structures and microcirculation [20]. In particular, OCTA for the first time isolates and visualizes the choriocapillary network in vivo, with information on the blood flow condition. Previous studies evaluating patients with chronic CSC by OCTA have reported areas with no pixel values in the choriocapillary layer at one week after HD-PDT treatment, implying hypoperfusion or even non-perfusion, without precise quantification [23,29].

Our findings related to the CC are in agreement with these previous histological and ICG angiographic observations [14,15]. In fact, in this study, the analysis of the OCTA scans by the binarization method showed a progressive increase of the non-perfused component during the follow-up after the treatment. In parallel, the luminal perfused component decreased and mostly at one week. Moreover, the ratio between the perfused luminal portion and the total area of the CC demonstrated a decrease in the first one hour after HD-PDT, with the restoration of the baseline values after one month. These findings reflect the results we previously found on a different cohort of chronic CSC eyes analyzed with a different method [23]. Again, we found that the choriocapillary vascular density was significantly reduced one week after HD-PDT. Moreover, we could not find any changes in the CC of the fellow non-treated eyes as an indirect confirmation of the direct effect of the PDT at this level. All these findings lead to believe that PDT works by producing a short-term and temporary choriocapillary hypoperfusion.

Our results showed a significant reduction of subfoveal CRT and CHT after HD-PDT from the baseline to one month starting one week after the treatment. However, these changes were not linearly correlated. Previous studies reported similar effects of PDT on the CHT [30], but the structures involved had not been determined yet. Our results by means of the binarization method showed that both LA and IA decreased in size after HD-PDT, with a final greater reduction by the LA.

In particular, the vascular component measured on the EDI-SDOCT scan increased one hour after the treatment. This might be the manifestation of a rebound effect and the inflammatory response of the tissues irradiated before the occlusion, as always observed after a laser treatment. The luminal component then significantly decreased one week and one month after HD-PDT. In addition, the ratio between LA and total structure demonstrated a progressive statistically significant reduction of the vascular component after the HD-PDT. Because the luminal areas decreased, we can postulate a reduction in the number of vessels and/or their diameter.

Our results are consistent with previous studies that reported a reduction of outer choroidal vascular dilation after half-dose PDT [31]. However, we demonstrated a reduction of both the choroidal vascular and extravascular components, indicating that a vascular decongestion, or closure, together with interstitial fluid reduction may be the tissue effect of HD-PDT on the choroid. In contrast, it has been observed that both choroidal luminal and stromal components remain unchanged in eyes with CSC after anti-VEGF therapy [32]. This lack of choroidal response may explain the superiority shown by PDT with respect to anti-VEGF therapy in the treatment of chronic CSC [33].

There are several limitations to our study. First, the small sample size and the use of fellow eyes as a control group do not allow definitive conclusions. Second, the presence of subretinal fluid in the analyzed area, whose quantity changed during the follow-up, could have interfered with the reflectivity signal from the underling choroidal structures. Third, we are aware of the intrinsic limits of the technology that we used for imaging choroidal vasculature. The SD-OCT system has more sensitivity loss with depth compared with swept-source systems, and thus, may be less suitable to visualize the CC as it is beneath the highly scattering RPE. Furthermore, both the binarization method and the slab selection might have influenced the results [34,35,36]. We selected the local thresholding Niblack method among the several different binarization methods because it is a publicly accessible software, and it was already previously applied for the study of the choroid in different conditions. A local thresholding method can be a fair solution to compensate for artifacts that create small regional variations in image brightness (e.g., light backscattering on SD-OCT or shadowing from vitreous bodies on OCTA) which, however, can still affect all the measurements. Finally, we selected the automated slab for CC, as it was previously validated in several studies [37].

## 5. Conclusions

In conclusion, we studied the effect of HD-PDT on the choroid of chronic CSC patients using SD-OCT and OCTA. Our results suggest that PDT induces a temporary relative reduction of the luminal components of the CC and the choroid, which return to the baseline status after one month from the treatment. However, after one month the choroid is thinner, hence, with a significant reduction of both luminal and interstitial components. Future studies with more sophisticated means of analysis of tomographic findings and larger sample size might provide further insight into the pathophysiology of CSC and its response to treatments.

## Figures and Tables

**Table 1:** Mean changes in central retinal thickness (CRT) and central choroidal thickness (CHT) after half-dose photodynamic therapy.

	CRT (SD) µm	*p*-Value	CHT (SD) µm	*p*-Value
Baseline	324.5 (92.6)		407.2 (97.2)	
1 hour	314.9 (69.3)	0.28	407.1 (103.6)	0.65
1 week	275.4 (50.2)	0.0002 *	362 (92)	0.034 *
1 month	252.5 (50.1)	<0.0001 *	341.5 (98.2)	0.0004 *

CRT = central retinal thickness; CHT = central choroidal thickness; SD = standard deviation. * = statistically significant (Mann-Whitney test *p* < 0.05).

**Table 2:** Mean changes of the luminal, interstitial and total (luminal + interstitial) choroidal area before and after half-dose photodynamic therapy measured by the mean of the binarization method on enhanced depth imaging spectral domain optical coherence tomography (EDI-SDOCT) scans. Mean ratio between luminal part and total structure (luminal + interstitial).

	Luminal (SD) μm^2^	Interstitial (SD) μm^2^	Total (SD) μm^2^ (Luminal + Interstitial)	Mean Ratio between Luminal Part and Total Structure
Baseline	2022.5354 (657.9681)	397.42755 (1007.2404)	599.6811 (1631.5287)	33.4% *
1 hour	2056.8678 (639.0139)	3794.3039 (1025.0755)	5851.1718 (1625.2475)	35.3% *
1 week	2006.1363 (618.6754)	3896.5376 (1079.9393)	5902.6739 (1671.5518)	33.9% *
1 month	1979.7304 (576.2866)	375.68110 (100.71412)	5736.5315 (1544.2734)	34.5% *

EDI-SDOCT = enhanced depth imaging spectral domain optical coherence tomography; SD = standard deviation. * = statistically significant (Friedman test *p* = 0.0018).

**Table 3:** Mean changes of the luminal, interstitial and total (luminal + interstitial) choroidal area before and after half-dose photodynamic therapy measured by the mean of the binarization method on optical coherence tomography angiography (OCTA) scans. Mean ratio between luminal part and total structure (luminal + interstitial).

	Study Eye		Fellow Eye	
	Luminal (SD) μm^2^	Mean Ratio between Luminal Part and Total Structure	Luminal (SD) μm^2^	Mean Ratio between Luminal Part and Total Structure
Baseline	5525.0509 (167.9515)	61.1% *	5399.3506 (86.1201)	60%
1 hour	5521.4951 (159.6833)	61% *	5390.8995 (70.8264)	59.9%
1 week	5460.1108 (120.9765)	60.4% *	5409.8341 (83.7363)	60.1%
1 month	5480.0464 (133.3034)	60.6% *	5401.9271 (112.9344)	60%

OCTA = optical coherence tomography angiography; SD = standard deviation. * = statistically significant (Friedman test *p* = 0.029).
